# Electrochemical Aptasensor for the Detection of the Key Virulence Factor YadA of *Yersinia enterocolitica*

**DOI:** 10.3390/bios12080614

**Published:** 2022-08-08

**Authors:** Maria G. Sande, Débora Ferreira, Joana L. Rodrigues, Luís D. R. Melo, Dirk Linke, Carla J. Silva, Felismina T. C. Moreira, Maria Goreti F. Sales, Ligia R. Rodrigues

**Affiliations:** 1 CEB—Centre of Biological Engineering, Universidade do Minho, Campus de Gualtar, 4710-057 Braga, Portugal; 2 LABBELS—Associate Laboratory, 4710-057 Braga, Portugal; 3 Section for Genetics and Evolutionary Biology, Department of Biosciences, University of Oslo, 0316 Oslo, Norway; 4 CENTI—Center for Nanotechnology and Smart Materials, Rua Fernando Mesquita 278, 4760-034 Vila Nova de Famalicão, Portugal; 5 CITEVE—Technological Center for the Textile and Clothing Industries of Portugal, Rua Fernando Mesquita 2785, 4760-034 Vila Nova de Famalicão, Portugal; 6 BioMark-CINTESIS/ISEP, School of Engineering, Polytechnic Institute of Porto, 4219-015 Porto, Portugal

**Keywords:** cell-SELEX, biosensor, aptamer, *Y. enterocolitica*, adhesin, YadA, cyclic voltammetry, impedance spectroscopy, square wave voltammetry

## Abstract

New point-of-care (POC) diagnosis of bacterial infections are imperative to overcome the deficiencies of conventional methods, such as culture and molecular methods. In this study, we identified new aptamers that bind to the virulence factor Yersinia adhesin A (YadA) of *Yersinia enterocolitica* using cell-systematic evolution of ligands by exponential enrichment (cell-SELEX). *Escherichia coli* expressing YadA on the cell surface was used as a target cell. After eight cycles of selection, the final aptamer pool was sequenced by high throughput sequencing using the Illumina Novaseq platform. The sequencing data, analyzed using the Geneious software, was aligned, filtered and demultiplexed to obtain the key nucleotides possibly involved in the target binding. The most promising aptamer candidate, Apt1, bound specifically to YadA with a dissociation constant (*K_d_*) of 11 nM. Apt1 was used to develop a simple electrochemical biosensor with a two-step, label-free design towards the detection of YadA. The sensor surface modifications and its ability to bind successfully and stably to YadA were confirmed by cyclic voltammetry, impedance spectroscopy and square wave voltammetry. The biosensor enabled the detection of YadA in a linear range between 7.0 × 10^4^ and 7.0 × 10^7^ CFU mL^−1^ and showed a square correlation coefficient >0.99. The standard deviation and the limit of detection was ~2.5% and 7.0 × 10^4^ CFU mL^−1^, respectively. Overall, the results suggest that this novel biosensor incorporating Apt1 can potentially be used as a sensitive POC detection system to aid the diagnosis of *Y. enterocolitica* infections. Furthermore, this simple yet innovative approach could be replicated to select aptamers for other (bacterial) targets and to develop the corresponding biosensors for their detection.

## 1. Introduction

Conventional ways to diagnose bacterial infections include classical methods, such as culture and microscopy, serological assays (e.g., enzyme-linked immunoassay (ELISA) and chemiluminescence immunoassays) and, more recently, molecular methods (e.g., reverse transcription–quantitative polymerase chain reaction (RT-qPCR), fluorescent in-situ hybridization (FISH) and microarrays). However, each of these methods are associated with certain disadvantages related to affordability, portability, speed and accessibility. The World Health Organization (WHO) has defined the characteristics of good diagnostic tests, especially in resource-limited settings, as affordable, sensitive, specific, user-friendly, rapid and robust, equipment-free and deliverable to end-users [1]. Moreover, the emergence of antibiotic resistant bacteria has been established as a global threat to health. Due to this, early detection of pathogens in humans and animals is essential to provide adequate treatment [2]. Thus, methods which can fulfil all these needs, such as point-of-care (POC) devices, have become increasingly important. Numerous sensor-based POC workflows for diagnosis of bacterial infections are under development [3].

Bacterial infections caused by the *Yersinia* species can cause symptoms like diarrhea, septicemia, mesenteric lymphadenitis and reactive arthritis in humans [4]. To diagnose infections with *Yersinia enterocolitica*, classical methods like enzyme-linked immunosorbent assays and western blots are often used, but cross-reaction with other *Enterobacteriaceae* is common, thus making it very difficult to diagnose using serological methods [5]. Culture methods are also used, but the time needed is prohibitively long as they can take a few days to up to a week or more [6]. Molecular methods like RT-qPCR are used to detect the enterotoxin-producing *ail* gene and Yersinia adhesin A (*yadA*) gene of *Y. enterocolitica* [7,8] and are very accurate diagnostics [6,9]. However, in the context of POC solutions, these methods are relatively expensive, time-consuming and require access to specialized laboratory equipment and trained staff [10,11]. To overcome the disadvantages of conventional methods of diagnosing *Y. enterocolitica* infections, it is of utmost relevance to develop novel POC solutions.

Electrochemical biosensors based on immobilized biorecognition elements are one of the most popular and commercially successful groups of biosensors. The development of screen-printed planar electrodes, which are cheap, sensitive and capable of miniaturization, augments the appeal of these biosensors [12]. In the context of pathogen detection and diagnosis, electrochemical biosensors are highly advantageous as they have enabled rapid and multiplexed detection of pathogens, as well as their direct detection from samples without prior processing. Electrochemical detection platforms are usually low-cost and portable with wireless actuation and data acquisition formats. As a result, their use has been widely investigated for medical diagnostic, food and water safety, environmental monitoring and for biological-threat applications [13,14]. Therefore, electrochemical biosensors are being increasingly used for the detection of pathogenic bacteria and other targets [15,16,17,18].

In order to design a high-performance biosensor, it is crucial that its biorecognition element possesses excellent analyte selectivity [19]. For instance, it is worth considering aptamers, which are short oligonucleotides isolated in vitro from randomized libraries that can bind to specific molecules with high affinity [20]. They are excellent bioreceptor candidates, as they offer a number of advantages over antibodies, such as superior stability, versatility and lower production costs [3,21]. The iterative selection process by which an aptamer is isolated is called systematic evolution of ligands by exponential enrichment (SELEX). When living cells, such as live bacteria, are used to select aptamers that bind specifically to a single target, or to multiple targets, present on the membrane surface of the same type of cell, it is termed cell-SELEX. The main advantage of cell-SELEX is that aptamers can be selected towards their targets, which are in their native form [22]. Aptamers selected by cell-SELEX have been widely used as receptors in biosensors for the detection of bacteria [23,24,25,26].

So far, only one study reporting aptamers selected by cell-SELEX against *Y. enterocolitica* is available [27]. In that study, three aptamers were selected against cells harvested at different growth stages and were further characterized. However, concrete cell surface target molecules were not analyzed or identified. Moreover, those aptamers have not been further evaluated in detection systems. Electrochemical detection systems incorporating aptamers as sensory molecules have been developed for a number of other pathogens, including *Listeria monocytogenes* [28], *Escherichia coli* O157:H7 [29] and *Salmonella enterica serovar Typhimurium* [30], and have proved to be highly efficient [31].

Selection of a suitable target or analyte is also important to consider while designing a biosensor. When aptamers are selected towards a target by cell-SELEX, the target is usually exposed on the outer surface of the cell, such as a protein. Certain bacteria, such as *Y. enterocolitica*, assemble protein complexes called adhesins on their surface, which recognize specific molecular receptors of the host during colonization and are suitable markers for detection of the bacteria [32,33]. YadA from *Y. enterocolitica* is such an adhesin and is classified as a homotrimeric autotransporter [34], a class of adhesins that have been shown to enable many Gram-negative pathogens to interact with host extracellular matrix proteins, such as collagen, vitronectin, and fibronectin [35,36]. During an infection, YadA mediates the binding to epithelial and polymorphonuclear cells, and is also essential for autoagglutination [32].

In this work, aptamers with high affinity towards the important disease marker YadA of *Y. enterocolitica* were selected, for the first time, through a combination of cell-SELEX with high throughput sequencing (HTS) and bioinformatics analysis. The use of cell-SELEX enabled the selection of aptamers towards YadA, which was recombinantly expressed on *E. coli* surface. Subsequently, the best candidate aptamer (Apt1) was immobilized on gold screen-printed electrodes (Au-SPE) for the electrochemical detection of *E. coli* cells expressing YadA. Furthermore, the performance of this aptasensor was successfully validated resulting in a novel, sensitive and selective POC detection system, which has the potential to be used towards the diagnosis of *Y. enterocolitica* infections.

## 2. Materials and Methods

### 2.1. Bacterial Strains and Plasmids

The plasmid pASK_IBA2 containing the YadA gene from *Y. enterocolitica* WA-314 [37] was previously developed at Linke lab [38]. *E. coli* BL21 (DE3) (NZYTech, Lisbon, Portugal) was used as host for pASK_IBA2 YadA (herein referred as *E. coli* YadA), as well as for empty plasmid pASK_IBA2 (referred as *E. coli* IBA), that was used in control experiments. *Pseudomonas putida* S12 was obtained from ATCC (#700801).

### 2.2. SsDNA Library, Primers and Aptamer Sequences

A randomized ssDNA library with 50 nucleotides (nt) flanked on both sides by constant primer binding regions (5′-ATCCAGAGTGACGCAGCA-N50-AATGTCGTTGGTGGCCC-3′) was used for cell-SELEX. The PCR amplification step in cell-SELEX was performed using a forward primer (5′-ATCCAGAGTGACGCAGCA-3′), and a 5′ end biotin modified reverse primer (5′ biotin-GGGCCACCAACGACATT-3′). Primers and library were purified by high performance liquid chromatography (HPLC) (Invitrogen). The selected candidate aptamer Apt1 was labelled with fluorescein (FAM) for characterization experiments. Later, Apt1 was thiol-modified (Metabion, Steinkirchen, Germany) and purified by HPLC for assembling the biosensor.

### 2.3. Cell-SELEX

**Preparation of culture**. *E. coli* YadA was grown by inoculating 1% of an overnight pre-inoculum in 5 mL Lysogeny Broth (LB; 10 g L^−1^ tryptone, 5 g L^−1^ yeast extract, 10 g L^−1^ NaCl) (NZYTech, Lisbon, Portugal) supplemented with 5 µL ampicillin (100 mg/mL, NZYTech, Lisbon, Portugal), and shaken at 37 °C and 200 rpm. After approximately 3 h, when an optical density at 600 nm (OD_600nm_) of 0.7 was reached, anhydrotetracycline (aTc, 200 µg L^−1^, Acros, Geel, Belgium) was added to induce expression of YadA. The cells were grown for an additional 2 h. After this, 500 µL of the *E. coli* YadA culture was centrifuged (6000 rpm for 8 min) to remove the supernatant, washed twice in phosphate buffer saline (PBS) and finally suspended in 600 µL of selection buffer (SB). SB was prepared by supplementing PBS 1x, pH 7.4 (137 mM NaCl (NZYTech, Lisbon, Portugal), 2.7 mM KCl (Chem-Lab, Zedelgem, Belgium), 8 mM Na_2_HPO_4_ (ChemLab) and 2 mM KH_2_PO_4_ (Panreac, Barcelona, Spain)) with 1.4 mM of MgCl_2_·6H_2_O (VWR, Radnor, PA, USA). When counter selection cycles were performed, the culture (*E. coli* IBA) was suspended in a volume of 100 µL SB.

**Figure 1:**
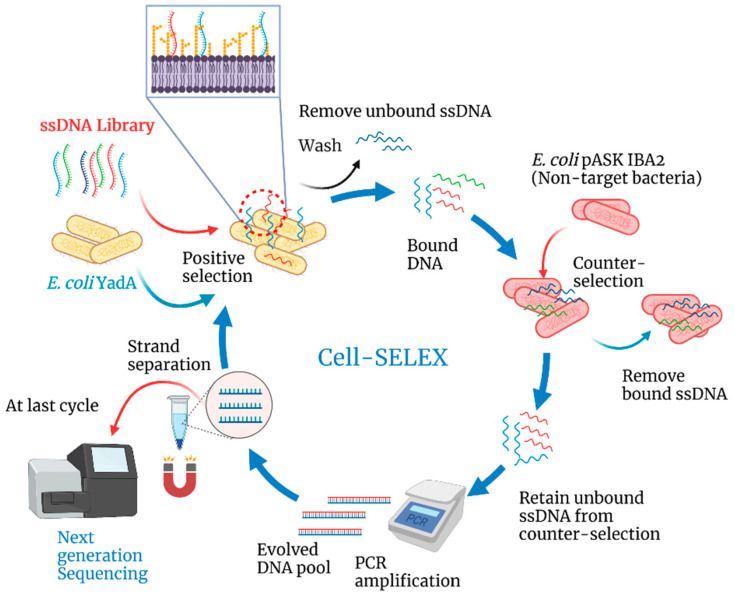
Schematic illustration of the steps in the cell-SELEX process to isolate an aptamer pool with high-affinity to YadA adhesin over 8 cycles of evolution starting with a ssDNA random library. Created with BioRender.com (accessed on 22 July 2022).

**Cell-SELEX**. The steps involved in each cell-SELEX cycle are schematically illustrated in Figure 1.

For the first cell-SELEX cycle, 2 nmol of ssDNA library was prepared by resuspending 20 µL of library (100 µM) in 180 µL SB followed by heating for 5 min at 95 °C and cooling at room temperature. The first cycle of selection took place by incubating the 2 nmol ssDNA library with the 600 µL *E. coli* YadA culture for 60 min at 37 °C with gentle shaking. Afterwards, the supernatant was removed (in the cases of counter selection cycles, the supernatant was retained and directly used for amplification by PCR), and the *E. coli* YadA cells were washed with a washing buffer (PBS 1x) to remove unbound ssDNA sequences. Next, the washed cells were heated for 5 min at 95 °C in 100 µL DNase-free water to elute the ssDNA sequences bound to the target YadA, and these sequences were amplified by PCR. During the second and subsequent cell-SELEX cycles, the ssDNA obtained from the preceding cycle was incubated with the target *E. coli* YadA cells. Further, with each cycle, the stringency was augmented by decreasing the time of incubation during the selection, increasing the number of washes after selection, and reducing the volume of cells used for positive selection (Table 1).

**Preparation of ssDNA**. After each cycle, PCR reactions were performed to amplify the eluted sequences, which were then used as the template for the next cycle. The PCR was performed using HiFi DNA Polymerase Master Mix (VWR). Primers (Section 2.3) were used at a final concentration of 0.2 μM. The conditions during the reaction were: 95 °C for 5 min, 15–35 cycles (see Table 1) of 30 s at 95 °C, 30 s annealing at 72 °C and 30 s extension at 72 °C, followed by a final extension of 5 min at 72 °C. A small amount of the PCR product (85 nt) was analyzed with a 3% agarose gel to verify the size of the dsDNA. The PCR product was purified using a MiniElute PCR purification kit (Qiagen, Germantown, MD, USA). 

The reverse-complement biotinylated DNA strands of the purified sequences were removed using streptavidin-coated magnetic beads (Dynabeads M-280 Streptavidin, Invitrogen, Carlsbad, CA, USA). To do this, the purified PCR product was heated for 5 min at 95 °C and cooled to separate the strands of dsDNA. An appropriate quantity of beads (1 mg beads/~10 µg dsDNA) were washed and resuspended in binding and washing (B&W) buffer 2× (10 mM Tris-HCl, pH 7.5, Fisher; 1 mM EDTA (Biochrom, Cambridge, United Kingdom) and 2 M NaCl) to a concentration of 5 µg/µL. An equal volume of the denatured dsDNA in distilled water was added to the beads and incubated for 15 min at room temperature with gentle rotation. The biotinylated reverse-complement strands bound to the streptavidin beads and were separated using a magnet for 2–3 min while the supernatant with the forward ssDNA was recovered and used in the next cell-SELEX cycle. 

**Sequencing and data analysis**. The ssDNA pool obtained after the final 8th cycle of cell-SELEX was outsourced sequenced (Stab Vida, Caparica, Portugal), using the lllumina Novaseq platform (short-read process) and 150 nt paired-end sequencing reads. The ssDNA pool was prepared by performing a PCR with primers complementary to constant regions of the random ssDNA library with an added overhang pertaining to a platform-specific sequence. Raw next-generation sequencing (NGS) data was analyzed using an alignment algorithm embedded in Geneious software 9.1.4 (Biomatters Ltd., Auckland, New Zealand; https://www.geneious.com/ accessed on 25 July 2022). Briefly, the sequencing reads were filtered and demultiplexed. During this analysis, constant primer binding regions were removed and sequences longer or shorter than 47–52 nt were discarded. The 10 oligonucleotide sequences which recurred most frequently were evaluated further. The mfold web server (version 3.0, http://www.unafold.org/mfold/applications/dna-folding-form.php accessed on 17 July 2022) was used to calculate the Gibbs free energy (∆G) and secondary structures of the sequences considering the primer and random regions, presenting an estimate of the optimal structure for each aptamer. The conditions used for the structure predictions were set according to the SB constitution (137 mM Na^+^ and 1.4 mM Mg^2+^ at pH 7.4) at 37 °C [39]. These sequences were aligned and the phylogenetic tree was constructed using the Tree Builder function in Geneious, that allowed the determination of the tree distances and sequence relatedness using a neighbor-joining model. 

### 2.4. Binding Experiments

To determine the dissociation constant (*K_d_*), 225 µL of *E. coli* YadA cells was incubated with increasing concentrations of FAM-labelled Apt1. The concentration values of aptamer samples ranged from 0 to 100 nM. Each aptamer sample was mixed with *E. coli* YadA cells suspended in SB up to a volume of 250 μL in Eppendorf tubes and incubated at 37 °C for 75 min with gentle agitation. After this, cells of each sample were retained and washed, resuspended in 250 μL of SB and then heated at 95 °C for 5 min to elute the FAM-aptamers which bound to the cells. After heating, the supernatant containing the eluted aptamers was recovered and transferred to a dark 96-well microtiter plate (Corning, Sigma) to measure their fluorescence intensity using a spectrophotometer from BioTek (Winooski, VT, USA), model Cytation 3 (excitation: 492 nm, emission: 518 nm). The *K_d_* of the aptamer-cell interaction was determined by plotting the mean fluorescence intensity of cells (*Y*), against the concentration of aptamer (*X*, nM) and the *B_max_* as the maximum number of binding sites, using a non-interacting binding sites model according to Equation (1) through GraphPad Prism (San Diego, CA, USA) (version 7.00 for Windows).

(1) $$Y=\frac{B_{max}\;X}{\left(K_{d}+X\right)}$$

### 2.5. Electrochemical Apparatus and Electrodes

The electrochemical measurements were performed with a potentiostat/galvanostat from Metrohm Autolab (Utrecht, Netherlands), PGSTAT320N, controlled by NOVA 1.11 software (Utrecht, Netherlands). Au-SPEs were purchased from DropSens (Asturias, Spain) (DS-C220AT). Each SPE had a platinum counter electrode, a reference electrode with silver electrical contacts and a gold working electrode 4 mm in diameter. Figure S1 shows a scanning electron microscope image of the bare Au working electrode surface. The Au-SPEs were interfaced in a switchbox from DropSens to facilitate their connection to a potentiostat. 

### 2.6. Development and Evaluation of the Aptasensor

The assembly of the Apt1-based biosensor included cleaning and immobilization of the isolated Apt1 onto the Au working electrode surface via thiol bonds, followed by the incubation of *E. coli* YadA with the aptamer. Thereafter, the sensitivity and selectivity of their interactions were assessed electrochemically.

#### 2.6.1. Assembly of the Aptasensor on an Au SPE

The water used for the biosensor development and validation was ultrapure Mili-Q laboratory grade (conductivity <0.1 µS/cm) and PBS was prepared from tablets from Amresco. Before modification of the bare SPE to assemble the biosensor, the working Au surface was cleaned by rinsing with absolute ethanol (99.5%, Panreac) and then washed thoroughly with ultrapure water to ensure that traces of ethanol and unwanted chemicals were removed. The active area of the electrode was calculated using the Randles-Sevcik equation [40]. The active area of the electrode before cleaning was found to be 0.202 cm^2^ and after cleaning was 0.215 cm^2^. The geometric area of the electrodes was 0.125 cm^2^. 

**Figure 2:**
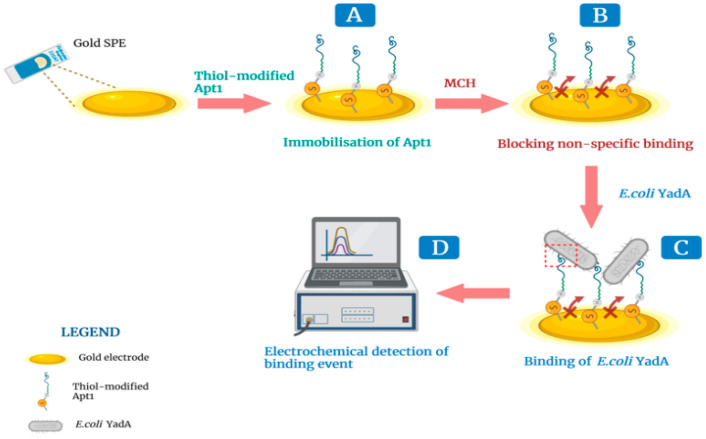
Schematic illustration of the sequential steps in the construction and working of an electrochemical biosensor for detection of the YadA adhesin. (**A**) Immobilization of Apt1 on the gold surface; (**B**) Blocking the inactive sites using 6-mercapto-1-hexanol (MCH) to prevent non-specific binding; (**C**) Affinity binding between Apt1 and YadA and (**D**) measurement of the electrochemical response after each step. Created with BioRender.com (accessed on 22 July 2022).

A simple, two-step method was used to assemble the biosensor. A stock solution (1 µM) of thiol-modified Apt1 prepared in phosphate buffer (5 mM MgCl_2_, Riedel-de Haen, Seelze, Germany), in PBS 1×, pH 7.4), was prepared in advance. The standard solution of Apt1 (0.5 µM) used in biosensor experiments, was prepared by diluting the stock solution. In the first step, the standard solution of Apt1 was deprotected by incubating it with dithiothreitol (DTT, 0.1 M, Sigma Aldrich, Steinheim, Germany) for 30 min at room temperature. DTT disrupts disulfide bonds and the deprotected Apt1 probe was then heated at 95 °C for 5 min to make it structurally flexible and interactive. Next, the Apt1 probe (5 µL) was added to the Au working surface and incubated for 2 h in a hydrated chamber at room temperature, where it became immobilized onto the Au surface via thiol groups at its 5′end (Figure 2A). The electrode surface was then washed with ultrapure water several times to remove any free Apt1 probe. In the second step, the Au surface was blocked by incubating the surface with 6-mercapto-1-hexanol (MCH, 5 mM, TCI Chemicals, Zwijndrecht, Belgium) for 2 h, at room temperature (Figure 2B). This step was critical to prevent interactions between biomolecules in a sample and the Au surface [41]. The construction of the aptamer-based biosensor was complete.

In general, during the detection of YadA, a solution of inactivated *E. coli* YadA in buffer was added (≈5 μL) to the Au working surface and incubated for 30 min (Figure 2C). To prepare the inactivated cells, 50 mL of *E. coli* YadA (7.0 × 10^7^ CFU mL^−1^) grown in LB was centrifuged (6000 rpm for 8 min) and the supernatant discarded. Subsequently, 10 mL of 70% ethanol was added to the cell pellet for 5 min. Next, the ethanol was thoroughly removed and the inactivated *E. coli* YadA cells were resuspended in 50 mL of PBS 1× (pH 7.4). After incubation, the electrode was washed with ultra-pure water to remove any non-bound bacterial cells. This was followed by the addition of 80 µL of the redox probe onto the Au working surface before initiating electrochemical impedance spectroscopy (EIS) and cyclic voltammetry (CV) assays to assess the level of binding between Apt1 and YadA (Figure 2D). All changes occurring at each step of the aptasensor assembly were also followed up by EIS and CV assays. The purpose of electrochemical procedures was to monitor the effectiveness of the Au surface modifications by recording EIS Nyquist plots and cyclic voltammograms of a typical redox probe.

#### 2.6.2. Evaluation of the DNA Aptasensor in Standard Solutions

To evaluate the sensitivity of the aptasensor, six standard solutions of the inactivated *E. coli* YadA were prepared by serial dilutions of the stock solution. Less concentrated standards were obtained by accurate dilution of the previous solution in one of the following buffers, as needed: 1 mM PBS 1× (pH 7.4), 1 mM 2-(*N*-orpholino) ethane sulfonic acid and 4-morpholine ethane sulfonic acid (MES) monohydrate (98%, AppliChem, Darmstadt, Germany) (pH 5.0). The concentrations of the standard solutions ranged from 7.0 × 10^4^ to 7.0 × 10^7^ CFU mL^−1^. The Au working electrode surface of aptasensors were incubated with each of the *E. coli* YadA standard solutions, for 30 min at 37 °C in a humid environment. Each assay was followed up with square wave voltammetry (SWV) together with EIS to assess the electrochemical properties. The sensitivity of the aptasensor was quantified through calculation of the limit of detection (LOD). The LOD was the concentration corresponding to X + 3σ, where X was the average value of the EIS or SWV blank signals (obtained in the absence of *E. coli* YadA) and σ the known standard deviation of EIS or SWV blank signal consecutive readings.

The selectivity of the aptasensor was established by a competitive assay between *E. coli* YadA and two other non-related bacteria, *P. putida* S12 and *E. coli* IBA. Selectivity studies were conducted by assessing, in SWV, the electrochemical response of the biosensor to 7.0 × 10^6^ CFU mL^−1^ *E. coli* YadA as a single analyte, and comparing this to the response of the biosensor to a mixed culture of *E. coli* YadA and *P. putida* S12 (both at a concentration 7.0 × 10^6^ CFU mL^−1^)*,* and, similarly, to a mixed culture of *E. coli* YadA and *E. coli* IBA. All cultures were prepared in MES buffer, pH 5.0. The incubation time of each test culture with the Au working surface was set to 30 min, i.e., the same period used in the sensitivity evaluations of the biosensor. These assays were performed in duplicate on the same day. In addition, the incubations were carried out at 37 °C in a humid environment. In each case, the decrease in peak current from the *I* value measured for a blank solution (MES buffer) was calculated as Δ*I*. The Δ*I* values of the mixed cultures were expressed in terms of percentage of the Δ*I* corresponding to the pure *E. coli* YadA.

Statistical analysis was performed to understand the effect of the non-target strains on the biosensor response, with the help of a one-way ‘analysis of variance’ (ANOVA) using the GraphPad Prism software.

### 2.7. Electrochemical Measurements

The electrical properties of the modified electrode surfaces were evaluated by CV, EIS and SWV assays, carried out in a redox probe solution of 5.0 × 10^−3^ M [Fe (CN)_6_]^3−^ and 5.0 × 10^−3^ M [Fe (CN)_6_]^4−^, prepared in PBS 1× buffer, pH 7.4. To prepare the redox probe, potassium hexacyanoferrate-III(K_3_ [Fe (CN)_6_]) and potassium hexacyanoferrate-II (K_4_ [Fe (CN)_6_]) trihydrate were obtained from Riedel-de Haen. All electrochemical assays were performed in triplicate or duplicate. CV assays were made for scanning potentials from −0.5 to +0.5 V, at a scan-rate of 50 mV/s. EIS assays were performed at an open circuit potential, using a sinusoidal potential perturbation with an amplitude of 0.01 V, and 50 data points, logarithmically distributed over 0.1–100,000 Hz frequency range. EIS data is commonly fitted to equivalent circuits of resistors and capacitors, such as the Randles’ equivalent circuit, which was also used in the present case. SWV assays were made for a potential range from −0.2 to +0.8 V, at a frequency of 25 Hz, and with a step height of 50 mV.

### 2.8. Statistical Analysis

In this study, a one-way ANOVA, that is a statistical analysis tool which compares the means of three or more independent groups to determine if there is a statistically significant difference between the corresponding population means, was used to determine if there was such a difference between the electrochemical responses of the three groups of cultures used in the selectivity assays. Data were expressed as mean ± standard error of the mean (SEM) of the three independent experiments. One-way ANOVA with Tukey’s post-test was used for comparison of the three normally distributed groups. A significance level of 95% was considered. Differences with *p*-value below 0.05 were considered statistically significant.

## 3. Results and Discussion

### 3.1. Selection of Aptamers against YadA through Cell-SELEX

The use of the pathogenic strain *Y. enterocolitica*, which naturally expresses YadA, as the target for selection of aptamers using cell-SELEX was avoided for safety reasons. Instead, recombinant YadA was expressed on the surface of a non-pathogenic *E. coli* BL21 DE3 host. This approach simplified implementation of the cell-SELEX as compared to other studies, which used the pathogenic bacteria directly, increasing workplace hazards.

In the first cell-SELEX cycle, a positive selection was performed. The initial ssDNA library was incubated with live *E. coli* YadA cells to attain the largest possible variety of ssDNA oligonucleotides binding to the target cells. From the second cycle onwards, bound sequences recovered from the previous cycle were incubated with the target cells for positive selection. After each incubation cycle, the ssDNA molecules bound to the target cells were eluted and amplified by PCR to be used in the next selection cycle.

During the 5th and 7th cycle, the target *E. coli* YadA cells were replaced by a negative control, *E. coli* IBA cells, for counter-selection, i.e., to eliminate any sequences recognizing surface molecules common to both strains. A total of 8 cycles were performed, as this was found to be optimal in other recent reports [42,43,44]. PCR results from each selection cycle were monitored by gel electrophoresis, showing that the band for the enriched ssDNA pool had the same size of the initial library with about 85 nt (*data not shown*). The pool of ssDNA from the final 8th cycle was sequenced through the Illumina Novaseq platform.

### 3.2. Identification of Aptamer Candidates Aided by Bioinformatics Tools

NGS was used to sequence the final aptamer pool generated through cell-SELEX. The use of NGS for this purpose is highly advantageous over conventional Sanger DNA sequencing, since the number of possible reads can be increased from a few to millions. Using NGS, there is a much greater possibility of selecting highly specific aptamers from among those generated by cell-SELEX [39,45]. Further, when high throughput sequencing is combined with bioinformatics analysis, other information, besides a large pool of potential aptamer candidates, can be derived, such as the number of total reads, frequencies of every unique sequence and the rate of molecular enrichment [46].

Therefore, the final pool of ssDNA was sequenced by NGS and the results were analyzed using Geneious. From 6,692,236 total sequences retrieved, 1,427,544 sequences were obtained after an initial filtration, removal of the adapter and constant primer binding region and length filtration (47–52 nt). The 10 most frequent oligonucleotide sequences were retrieved by Geneious refining and are presented in Table 2 (exhibiting only the randomized region).

**Figure 3:**
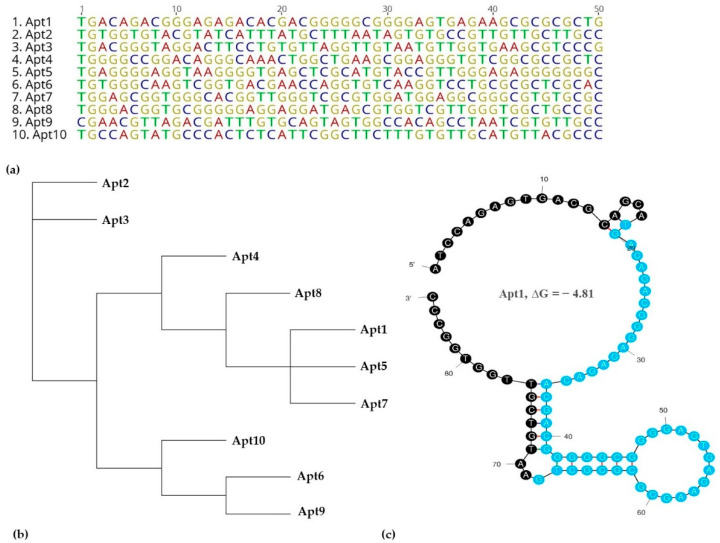
Primary structure, phylogenetic tree and secondary structure of the selected aptamers: (**a**) Multiple sequence alignments of the random region from the ten most repeated oligonucleotide sequences obtained after NGS. (**b**) Phylogenetic analysis of the ten aptamer sequences was determined using the Tree Builder function in Geneious software. (**c**) Predicted secondary structure for aptamer candidate Apt1 that was selected for further in vitro characterization experiments. The presented predicted secondary structure was the one with lowest ΔG, i.e., the highest stability using the temperature 37 °C, 137 mM Na^+^, and 1.4 mM Mg^2+^, as calculated using the mfold web server. The randomized region is presented in blue.

These ten sequences were aligned for homology within their variable core region (Figure 3a,b).

The analysis of the variable core nucleotide sequences was performed regarding secondary structure which was predicted using the mfold web server to observe their folding characteristics, considering the primer and random regions (Figure 3c and Table S1). For instance, Apt2 had three loops in its structure, Apt1, 3, 5, 6, 8 and 10 had four loop structures, Apt4 and 9 had five loops and Apt7 had six loops. The nucleotides which form the loop structures were further investigated. The N50 regions of the aptamers, when aligned, showed some conserved sequences, such as ‘GTTG’ found in the loop regions of Apt2, 3, 5, 8, 9 and 10. This suggested that these aptamers recognized the same target. Other motifs included ‘TGAC’ found in the loop structures of Apt1, 3 and 6; ‘GAAGCG’ in loop structures of Apt1 and 4; ‘GGAG’ in loop structures of Apt1, 4, 5 and 7 and ‘TGGG’ in loop structures of Apt 4, 5, 6, 7 and 8.

From among these 10 aptamer sequences, Apt1 was chosen as the candidate aptamer based on its pool repeatability, as well as on the presence of different conserved sequences in its secondary structure. The sequence of Apt1 was synthesized with a FAM label to be used in the subsequent characterization experiments.

### 3.3. Determination of Apt1 Affinity to YadA

**Figure 4:**
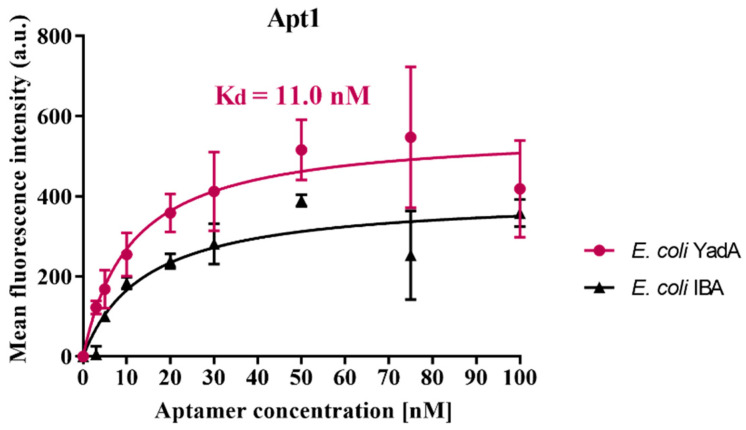
Determination of the equilibrium dissociation constant of candidate aptamer Apt1. The binding curves of aptamer Apt1 with *E. coli* YadA and *E. coli* IBA cells (control), respectively, is shown. The cells were incubated with increasing concentrations of FAM-labelled aptamers and assessed by fluorescence spectrophotometer. Equilibrium dissociation constant (*K_d_*) (nM) was calculated using GraphPad Prism 7, under the non-linear fit model, one-site non-competitive binding to fluorescent population ratio at given aptamer concentrations. a.u = arbitrary units.

In order to evaluate the binding affinity between the aptamer candidate Apt1 and YadA, the *K_d_* value was calculated using Equation (1). Figure 4 shows the aptamer’s binding saturation curves with the target *E. coli* YadA cells and control cells (*E. coli* IBA).

Apt1 has a high affinity to the *E. coli* YadA indicated by a low *K_d_* of 11 nM. The only other known study which used cell-SELEX to select aptamers against *Y. enterocolitica* reported the *K_d_* values for three final aptamers as 37.93 nM, 74.96 nM and 73.02 nM [27]. Based on the affinity studies in the present case, it can be argued that Apt1 would bind with higher affinity to *Y. enterocolitica* (YadA) than the aptamers generated in [27]. Similar studies, which used cell-SELEX to isolate aptamers towards pathogenic bacterial targets, also reported binding affinities of aptamer-target interactions (*K_d_* values) in the low nanomolar range [44,47,48]. However, a limitation of the experiment performed to calculate the *K_d_* presented in this work is that the interaction studied was between *E. coli* YadA and Apt1 rather than the interaction between pure YadA protein with Apt1, so the true *K_d_* value was probably higher than 11 nM. By extension, the true binding affinity was probably lower. Nevertheless, it could be concluded that Apt1 demonstrated selective binding properties towards YadA, making it a suitable candidate aptamer for validation as a biorecognition element in a system to detect YadA.

### 3.4. Electrochemical Evaluation of Aptasensor Assembly

EIS is the most common technique used in impedimetric biosensors. Its response can provide useful information about chemical modification made to the Au-SPE surface which alters its electrical features. EIS data provides insight into the charge transfer from solution to the electrode surface, solution resistance, as well as diffusion transport of species to and from the bulk solution and double layer capacitance formation [49]. The redox probe [Fe (CN)_6_]^3−^/[Fe (CN)_6_]^4−^ at pH 7.4, was chosen to monitor the electron transfer properties, due to its fast electron transfer rate [50]. 

**Figure 5:**
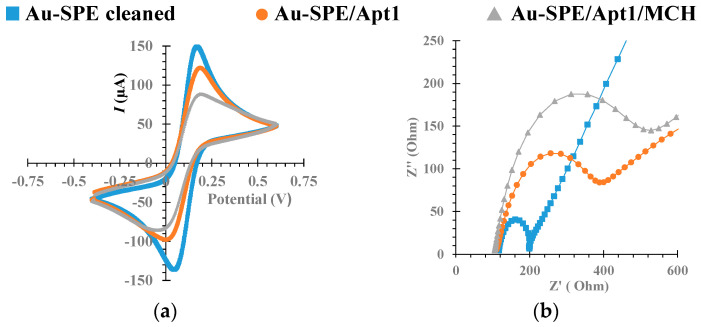
Electrochemical assays for construction of the biosensor by Au surface modification, in 5.0 × 10^−3^ M [Fe (CN)_6_ ]^3−^ and 5.0 × 10^−3^ M [Fe (CN)_6_]^4−^ solution, prepared in phosphate buffer, pH 7.4. Cyclic voltammetry (**a**) and electrochemical impedance spectroscopy Nyquist plot (**b**).

EIS and CV were used to monitor each step of the aptasensor development. The corresponding Nyquist plot and voltammogram are shown in Figure 5.

CV analysis is shown in Figure 5a. When compared to the clean surface, the Au-SPE modified with Apt1 (Au-SPE/ Apt1), and then MCH (Au-SPE/Apt1/MCH), showed decreased cathodic/anodic peak currents, due to an increased charge-transfer resistance at the Au-SPE surface. These results suggested that the Apt1 was successfully assembled on the Au surface.

The corresponding EIS measurements were consistent with CV data, and were characterized using Randles’ equivalent circuit, which fit the physicochemical process occurring at the Au electrode surface, yielding Nyquist plots that provided insight into the system dynamics. The Nyquist plot, shown in Figure 5b, included a semicircle portion, the diameter of which corresponded to the electron charge-transfer resistance (Rct), which was inversely proportional to the rate of electron transfer [51], and a linear portion, known as Warburg impedance, which represented a diffusion limited process [52]. Overall, the bare Au electrode showed a small semi-circle domain, which suggested a mostly mass diffusion limiting step of the electron transfer process. Immobilization of the Apt1 probe on the clean Au SPE surface increased the Rct considerably. This observation was consistent with an increased electron transfer resistance at the surface due to electrostatic repulsion between the negatively charged phosphate backbone of single stranded Apt1 and a negatively charged redox couple hindering the electrode transfer event [53]. After subsequent blocking of the free Au surface with MCH, the diameter of the semicircle of the Nyquist plot increased further.

Figure S2 shows the corresponding Bode phase plot and Randles’ equivalent circuits. The circuit in the Nyquist plot was simulated and adjusted to obtain the corresponding Randles’ equivalent circuit which led to two different equivalent circuits. The circuit obtained for the cleaned Au surface corresponded to Randles’ [R(RC)W]. After immobilization of the Apt1 probe on the Au surface, a more complex circuit was obtained with two-time constants that can be attributed to the presence of the thiol and base pairs of the aptamer sequence.

Thus, the EIS and CV data corroborated each other, and confirmed the occurrence of chemical changes at the Au surface for each step of the biosensor assembly. Furthermore, the assembly steps were found to be highly reproducible (relative standard deviation (RSD) was 3.66%, *n* = 3 in CV and 2.5% in EIS) and displayed excellent repeatability (RSD was 1.3%, *n* = 3 in CV and 1.5% in EIS).

### 3.5. Optimization of Experimental Conditions

Relevant experimental conditions were optimized to enhance the performance of the aptasensor. During the process of the aptasensor assembly, several Apt1 concentrations were assessed to improve aptamer immobilization on the working Au modified surface. The concentrations of Apt1 evaluated were 1, 0.5, 0.05 and 0.005 µM. The electrochemical responses in CV revealed that the peak current values were higher when the Au surfaces were modified with an Apt1 concentration of 0.5 µM (*data not shown*). Therefore, a concentration of 0.5 µM Apt1 was selected for the aptasensor assembly.

Mercaptosuccinic acid (97%, Sigma Aldrich), MCH and 3-mercaptopropionic acid (Sigma) were assessed as blocking agents. Only MCH showed a decrease in peak current values in CV voltammograms over those measured, after immobilization of Apt1. This could be attributed to the more efficient binding of MCH to the free Au surface when compared to the other thiol-based compounds. Hence MCH was used as the blocking agent for all pertinent studies.

### 3.6. Electrochemical Detection of E. coli YadA

**Figure 6:**
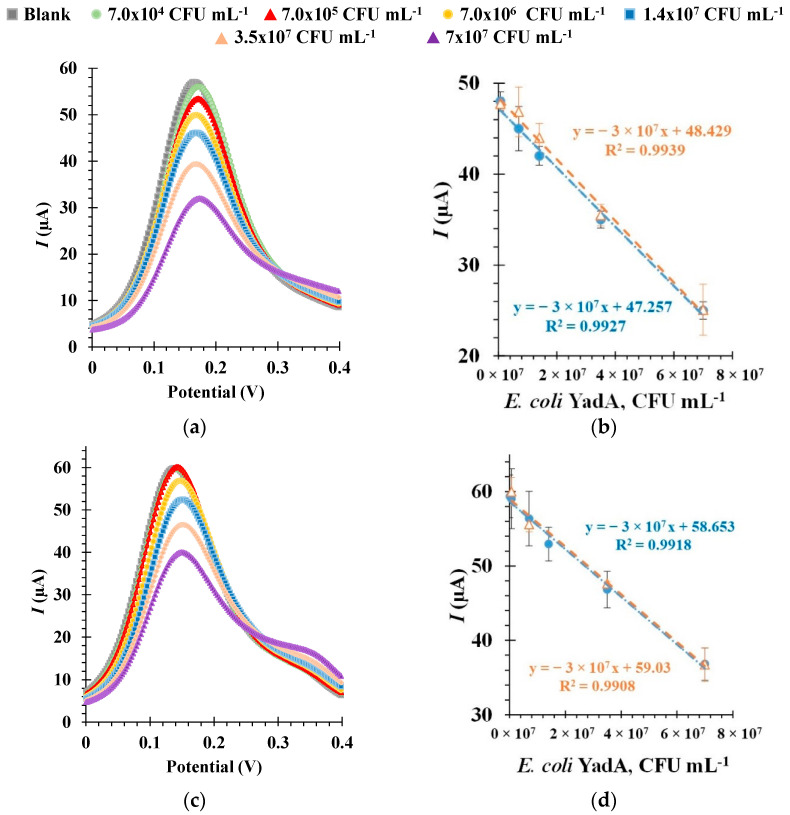
Square wave voltammetry (SWV) measurements (**a**,**c**), and the corresponding calibration curves (**b**,**d**), in 5.0 × 10^−3^ M [Fe (CN)_6_]^3−^ and 5.0 × 10^−3^ M [Fe (CN)_6_]^4−^ in phosphate buffer, pH 7.4. For these studies, the aptasensors were immobilized with Apt1 and incubated sequentially with varying concentrations of *E. coli* YadA cells maintained at pH 5.0 (**a**,**b**) or at pH 7.4 (**c**,**d**).

The analytical performance of the aptasensor was evaluated through calibration curves based on data derived from SWV measurements, as shown in Figure 6. Furthermore, the selected optimal experimental conditions were used during these analyses. SWV was chosen for this analysis as it has many advantages over other voltammetric techniques, such as greater sensitivity, a short analysis time and the ability for significant reduction of capacitance currents [54]. During the evaluation, increasing concentrations of *E. coli* YadA were incubated at the electrode surface for a set time, and the resulting current intensity (*I*) values obtained with the redox probe were measured against the *E. coli* YadA concentrations.

It is well known that the pH of the target buffer solution has an influence on ligand-target binding and, by association, on the performance of the electrochemical sensor [55]. So, to study the effect of pH on the detection of *E. coli* YadA by the aptasensor, the *E. coli* YadA standards were suspended in a buffer at pH 5.0 (MES) in a first study, and at pH 7.4 (PBS) in a second study, prior to their incubation on the electrode surface.

In general, the increasing *E. coli* YadA concentrations were revealed by decreasing peak currents (Figure 6a,c). This behavior was attributed to increasing charge transfer resistance with increasing concentration of *E. coli* YadA in each standard solution. The linear plots were made by correlating peak current values and *E. coli* YadA cell concentrations (Figure 6b,d). The response of the biosensor at each pH in SWV was evaluated in multiple calibrations, two of which are represented as orange and blue plot lines. Each calibration was made with three independent sensors, on different days, and was found highly reproducible (RSD 1.55%, *n* = 3 in SWV). The repeatability of successive measurements during a calibration was also found to be excellent (RSD 1.4%, *n* = 3 in SWV).

Selected calibration data at pH 5.0 is shown in Figure 6a, b. As observed from the SWV voltammograms, a decrease in current intensity *I* of 1.09 µA, which corresponded to *E. coli* YadA concentration of 7.0 × 10^4^ CFU mL^−1^, indicated that the aptasensor was sensitive enough to detect YadA at the lowest concentration tested under pH 5.0. The corresponding linear plots (Figure 6b) showed a square correlation coefficient >0.99. A linear correlation could be established for a dynamic concentration range from 7.0 × 10^4^ CFU mL^−1^ to 7.0 × 10^7^ CFU mL^−1^. As each calibration was performed in triplicate, the standard of deviation of these assays was ~2.5%. The calculated LOD was 7.0 × 10^4^ CFU mL^−1^.

The SWV voltammogram measured at pH 7.4 (Figure 6c) indicated that at around 7.0 × 10^5^ CFU mL^−1^ the sensor reached a detection saturation, since the *I* did not decrease any further at 7.0 × 10^4^ CFU mL^−1^. The corresponding linear plots had a linear trend between 7.0 × 10^5^ and 7.0 × 10^7^ CFU mL^−1^ (Figure 6d) and showed a square correlation coefficient >0.99. The standard deviation of these assays and the LOD was also ~2.5% and 7.0 × 10^5^ CFU mL^−1^, respectively. Overall, the response of the aptasensor seemed more sensitive at pH 5.0, as there was a difference between the signals of the blank solution and *E. coli* YadA concentration of 7.0 × 10^4^ CFUmL^−1^. At pH 7.4 this concentration yielded the same signal as the blank.

**Figure 7:**
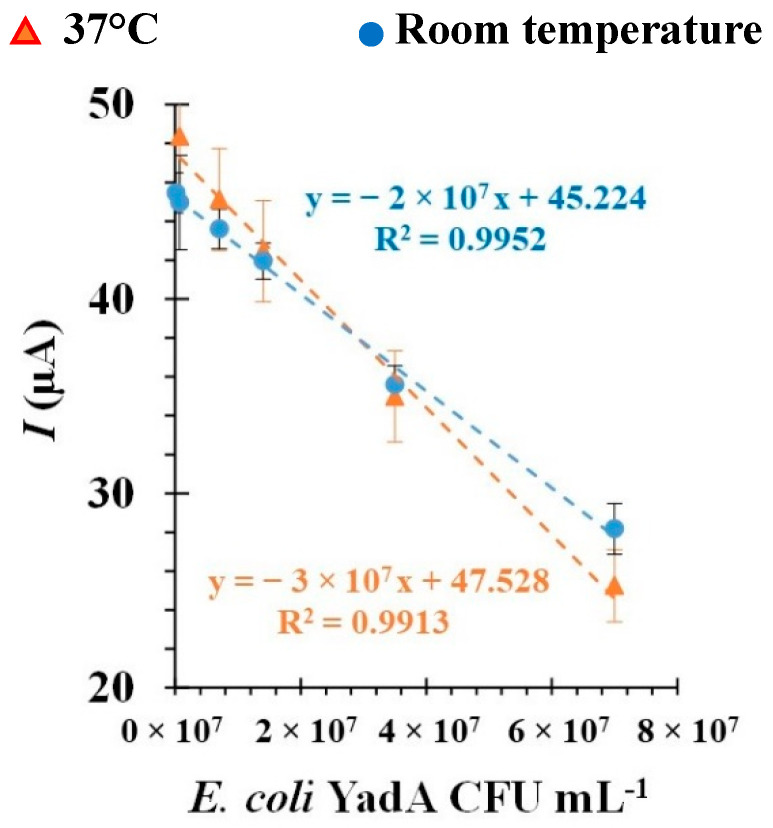
Comparison of the calibration curves in 5.0 × 10^−3^ M [Fe (CN)_6_]^3−^ and 5.0 × 10^−3^ M [Fe (CN)_6_]^4−^ in phosphate buffer, pH 7.4, with different concentrations of Apt1 at pH 5.0 incubated at 37 °C (orange) or at room temperature (blue).

Other factors that can influence the performance of the aptasensor include environmental conditions, such as temperature, as well as time of incubation during the detection step. Therefore, preliminary studies were performed to analyze the effect of these parameters. Following the usual analytical procedures, and at the selected optimal conditions, the incubation of biosensors with *E. coli* YadA cells was conducted at room temperature or 37 °C in a humid environment. From the results of the calibration, shown in Figure 7, it could be deduced that the biosensor showed slightly better sensitivity at 37 °C, owing to the higher slope value. Both linear plots showed a square correlation coefficient >0.99. Based on these results, all pertinent experiments were performed at 37 °C. Time of incubation, whether 30 or 45 min, did not herald any discernable changes during analysis of the biosensor performance (*data not shown*).

Most relevant studies which have endeavored to develop methods for the detection of *Y. enterocolitica*, have also reported an LOD of around 10^4^ CFU mL^−1^, similar to the present study (as shown in Table 3). One of these methods made use of magnetic beads coated in target-specific antibodies and nested PCR [56]. While other methods included a sandwich-type multiplex chemiluminescence immunoassay [57] and a biosensor, designed using single-walled carbon nanotubes immobilized with an antibody [58]. A surface plasmon resonance immunosensor detected *Y. enterocolitica* in a linear dynamic range of 10^2^–10^7^ CFU mL^−1^ [59]. Only one fairly recent study of an immunosensor equipped with graphene quantum dots reported a superior LOD of 30 CFU mL^−1^ [60]. Detection was rapid in all cases. However, the quality of the antibodies was a limiting factor affecting specificity of these methods [8]. On account of these limitations, aptamers are a good alternative to antibodies, as they exhibit significant advantages, including easier and cheaper production methods and better chemical stability [3]. It is worth noting that, while antibodies are generally reported to have superior affinity properties towards targets compared with aptamers, the LOD achieved with Apt1 was comparable, illustrating the applicability of the developed aptasensor for sensitive detection of *Y. enterocolitica* and in a POC setting, as per our objective. Various types of PCR [61] and ELISA [62,63] methods have been recently developed to detect *Y. enterocolitica* for diagnostic applications, but have the disadvantages of being time-consuming and/or requiring expensive instrumentation and highly-skilled operators. By contrast, the developed biosensor here described would be inexpensive to produce, easy to use and provide quick detection.

Overall, it is clear that the number of quality sensors and rapid POC-based studies, with the goal of detecting *Y. enterocolitica*, are low and immunological in nature. By contrast, the developed biosensor is a novel solution, and, in terms of performance, it showed sensitive and reproducible performance, as observed from the analysis of the SWV voltammograms and linear plots. The obtained results suggested that the biosensor, under optimal conditions, exhibited high sensitivity to YadA.

### 3.7. Selective Detection of E. coli YadA

**Figure 8:**
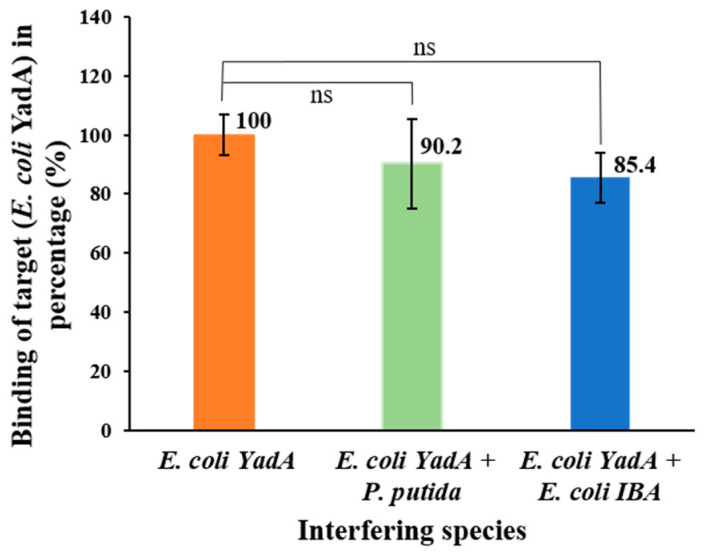
Selectivity behavior of the biosensor with Apt1 for *E. coli* YadA against *P. putida* and *E. coli* IBA after 30 min incubation and for the same redox probe previously tested. All bacterial strains were maintained at a concentration of 7.0 × 10^6^ CFU mL^−1^. One-way ANOVA indicated that the differences between group means were not statistically significant and denoted ns for *p* > 0.05, the *p* value was 0.34.

The selectivity study was conducted by evaluating any effect on the electrochemical response of the aptasensor by an interfering species in mixed cultures of the *E. coli* YadA and a non-target bacterial strain, using competitive assays. The interfering bacteria studied were *E. coli* IBA (which was the control strain, lacking YadA gene expressed in the plasmid) and *P. putida* S12 (also a non-pathogenic bacteria). Since it was a proof-of-concept study, the choice of non-target bacteria was focused on convenience and safety concerns. It was found that the presence of other bacteria, along with *E. coli* YadA, in equal concentrations had no meaningful effect on the *I* when compared to assays in the presence of *E. coli* YadA only (Figure 8).

To illustrate this, the percentage average deviation produced by each interfering species to the detection of *E. coli* YadA was shown in the bar chart to be 9.8 ± 15% for *P. putida* S12 and 14.6 ± 5% for *E. coli* IBA, when considering the detection of pure *E. coli* YadA as 100%. Overall, no significant interference was offered by the *E. coli* IBA and *P. putida* S12 to the binding between *E. coli* YadA and Apt1 in the mixed cultures, indicating selectivity of Apt1 toward YadA.

## 4. Conclusions and Future Perspectives

In this study, we have developed a simple and sensitive label-free electrochemical aptasensor for the detection of the adhesin YadA of *Y. enterocolitica.* A new DNA aptamer was selected by cell-SELEX, which demonstrated high affinity towards YadA. Subsequently, an Au-SPE was modified with the aptamer and the resulting aptasensor displayed high sensitivity and reproducibility, with an LOD of 7.0 × 10^4^ CFU mL^−1^ for *E. coli* YadA samples and it showed good selectivity in preliminary studies with mixed samples containing other bacterial strains. To further improve this work, a larger number of bacterial strains, including *Y. enterocolitica*, and real patient samples, should be tested to evaluate the sensitivity and feasibility of the biosensor in clinical applications.

In the future, the methods and workflow used to develop this aptasensor could also be used as a model to develop other biosensors for the detection of other bacterial targets. To enhance the applicability of this simple-to-produce biosensor to diagnostics, nanomaterials such as ligand-coated nanoparticles can be used for pre-enrichment of real samples. In addition, the availability of different aptamers from the NGS data, which were also characterized, and which present different binding sites for the detection of the same protein, could allow the design of more sensitive aptasensors. Furthermore, aptamers retrieved against proteins from other pathogens can be combined in the development of multiplexed aptamer arrays, for the simultaneous detection of multiple pathogens that may be present in real samples from patients.

## Figures and Tables

**Table 1:** Conditions used during the 8 selection cycles of cell-SELEX.

Cycles	*E. coli* YadA (µL)	Incubation (minutes)	Washes (After Selection)	PCR (Cycles)
1	500	60	2	35
2	500	60	2	30
3	500	60	3	30
4	400	50	3	30
5	400 (counter selection)	50	3	20
6	400	40	4	25
7	350 (counter selection)	35	4	15
8	350	35	4	20

**Table 2:** Selected oligonucleotide sequence candidates. The ten most frequent oligonucleotide sequences obtained after next-generation sequencing (NGS) of the 8th cell-SELEX cycle. Only the sequence corresponding to the random region is shown (primer binding sites are nor represented).

Aptamer	Sequences (5′–3′)	Copies
Apt1	TGACAGACGGGAGAGACACGACGGGGGCGGGGAGTGAGAAGCGCGCGCTG	6647
Apt2	TGTGGTGTACGTATCATTTATGCTTTAATAGTGTGCCGTTGTTGCTTGCC	4531
Apt3	TGACGGGTAGGACTTCCTGTGTTAGGTTGTAATGTTGGTGAAGCGTCCCG	1755
Apt4	TGGGGCCGGACAGGGCAAACTGGCTGAAGCGGAGGGTGTCGGCGCCGCTC	1443
Apt5	TGAGGGGAGGTAAGGGGTGAGCTCGCATGTACCGTTGGGAGAGGGGGGGC	1281
Apt6	TGTGGGCAAGTCGGTGACGAACCAGGTGTCAAGGTCCTGCGCGCTCGCAC	1214
Apt7	TGGAGCGGTGGGCACGGTTGGGTCGCGTGGATGGAGGCGGGCGTGTGCGC	1168
Apt8	TGGGACGGTGCGGGGGAGGAGGATGAGCGTGGTCGTTGGGTGGCTGCCGC	1080
Apt9	CGAACGTTAGACGATTTGTGCAGTAGTGGCCACAGCCTAATCGTGTTGCC	1023
Apt10	TGCCAGTATGCCCACTCTCATTCGGCTTCTTTGTGTTGCATGTTACGCCC	1012

**Table 3:** Examples of previous studies on the detection of *Yersinia enterocolitica* and the LOD achieved in each study.

Biorecognition Element	Detection Method	LOD	Reference
Yersinia-specific antibody; *yadA* gene	immunomagnetic separation and nested PCR	10^4^ to 10^7^ CFU mL^−1^	[56]
Y. enterocolitica-specific antibody	surface plasmon resonance-based immunosensor	10^2^ to 10^7^ CFU mL^−1^	[59]
Y. enterocolitica-specific antibody	sandwich chemiluminescent enzyme immunoassay	10^4^−10^5^ CFU mL^−1^	[57]
Y. enterocolitica-specific antibody	single-walled carbon nanotube-based biosensor	10^4^ CFU mL^−1^	[58]
Y. enterocolitica-specific monoclonal antibody	graphene quantum dots-based immunosensor	30 CFU mL^−1^	[60]
YadA aptamer	Cell-SELEX and electrochemical biosensor	10^4^ CFU mL^−1^	Present study

## Supplementary Materials

The following supporting information can be downloaded at: https://www.mdpi.com/article/10.3390/bios12080614/s1, Figure S1: Scanning electron microscope image of the bare Au working electrode surface. Figure S2: Bode phase plots and Randles’ equivalent circuit at biosensor assembly. Table S1: Predicted secondary structures of the other nine highest-copy aptamers.
