# Supplementary material for: Electrochemical Aptasensor for the Detection of the Key Virulence Factor YadA of Yersinia enterocolitica
Source: Biosensors (Basel). 2022 Aug 8;12(8):614. doi: 10.3390/bios12080614 (PMC9405658; doi:10.3390/bios12080614)
Supplement: Supplementary file 1 [file biosensors-12-00614-s001.zip › biosensors-1827548-supplementary.pdf]

Electrochemical aptasensor for the detection of the key virulence factor YadA of *Yersinia enterocolitica*

Maria G. Sande [1,2], Débora Ferreira [1,2], Joana L. Rodrigues [1,2], Luís D. R. Melo [1,2], Dirk Linke[3], Carla J. Silva [4,5], Felismina T. C. Moreira [1,2,6], M. Goreti F. Sales [1,2,6] and Ligia R. Rodrigues [1,2,*]

Corresponding author: Tel.: (+351) 253601978, Email: lrmr@deb.uminho.pt

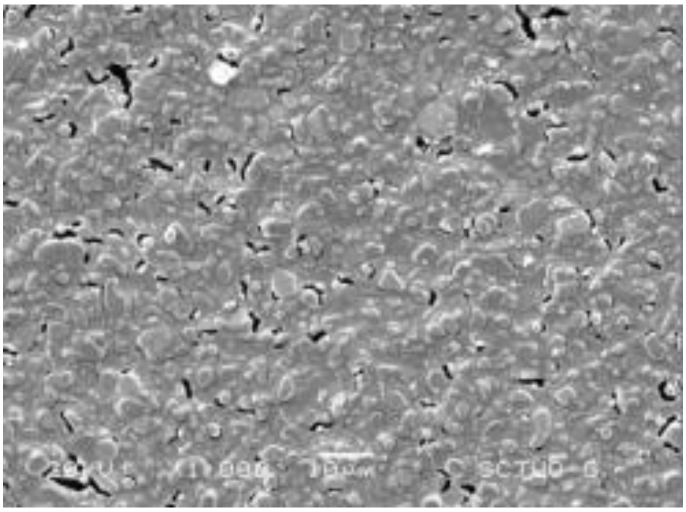

Figure S1. Scanning electron microscope image of the bare gold (Au) working electrode surface. (Image retrieved from Metrohm Dropsens https://www.dropsens.com/en/pdfs_productos/new_brochures/gold_electrodes.pdf)

Table S1. Predicted secondary structures of the other nine highest-copy aptamers. The presented predicted secondary structures were the ones with lowest ΔG, i.e., the highest stability using the temperature 37 °C, 137 mM Na^{+} and 1.4 mM Mg^{2+} (calculated using the mfold webserver). Random regions are presented in blue.

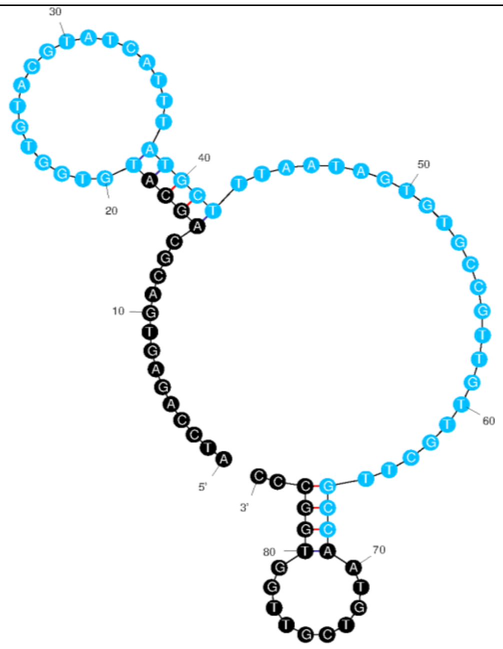 **Apt2, ΔG = −3.29**	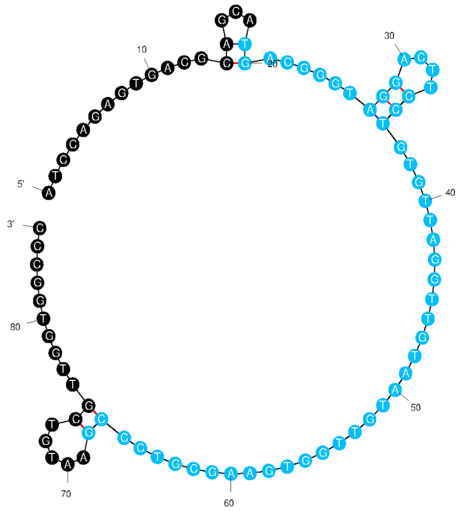 **Apt3, ΔG = −1.98**
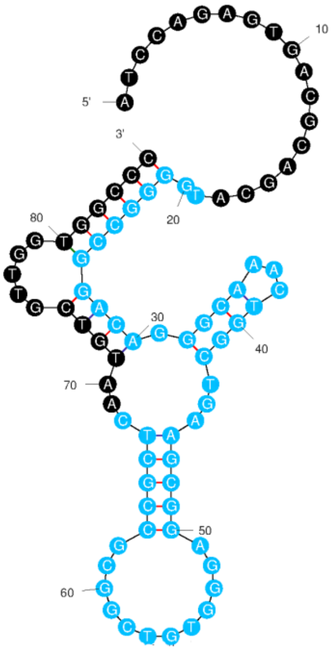 **Apt4, ΔG = −5.98**	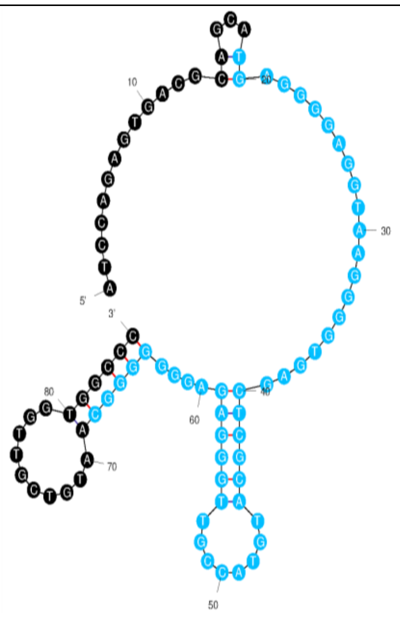 **Apt5, ΔG = −5.57**

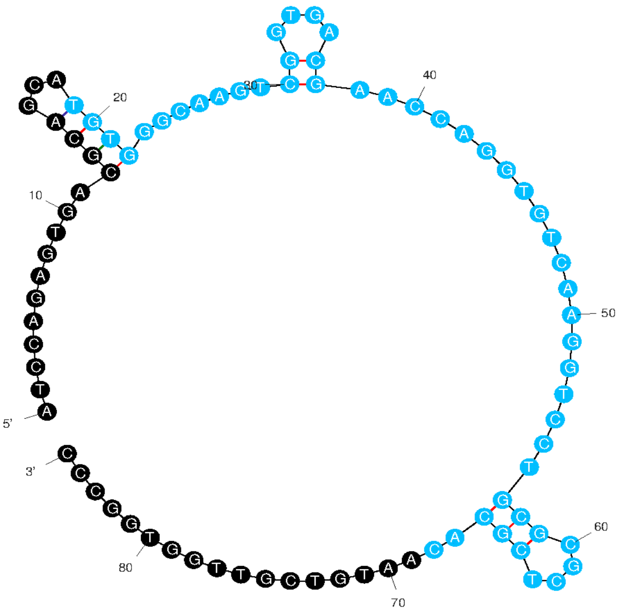

Apt6, ΔG = −5.29

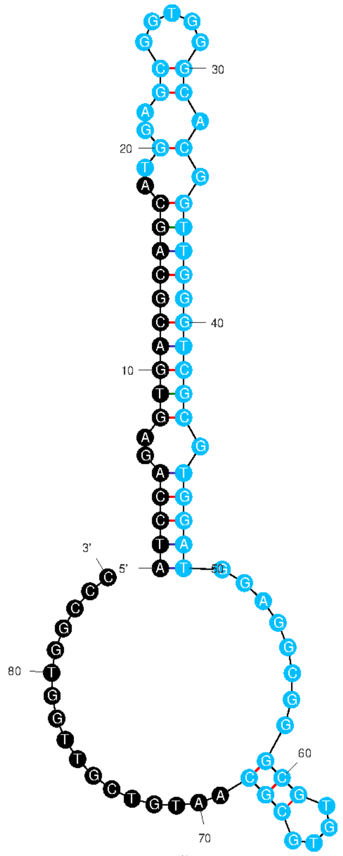

Apt7, ΔG = −7.11

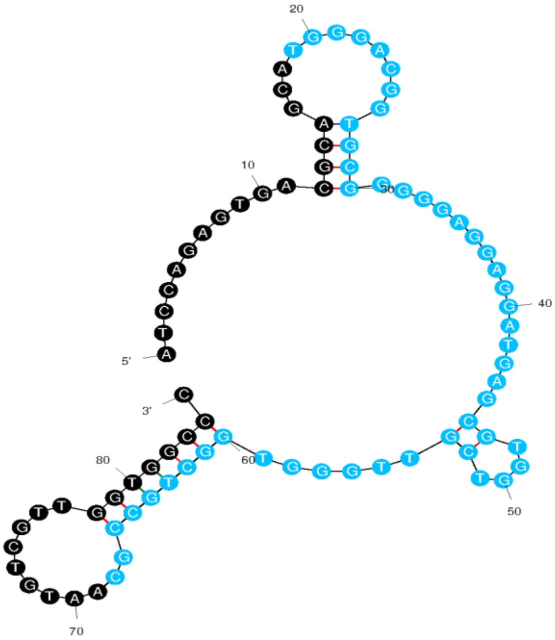

Apt8, ΔG = −5.75

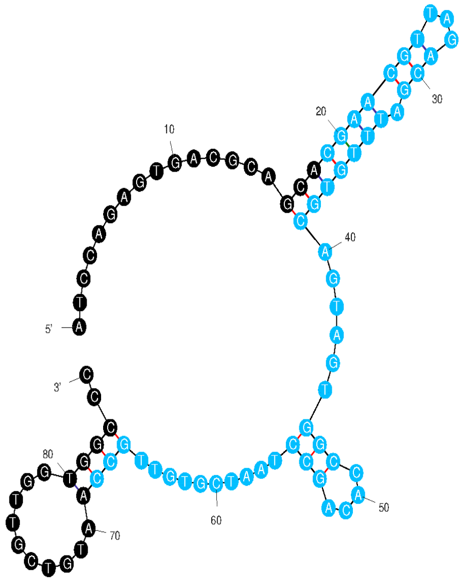

Apt9, ΔG = −7.51

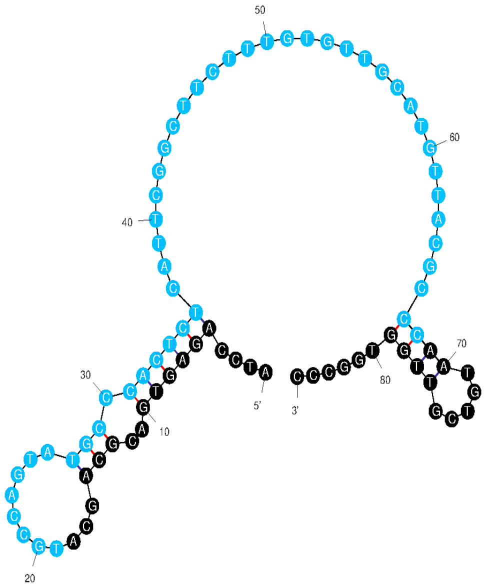

Apt10, ΔG = −5.56

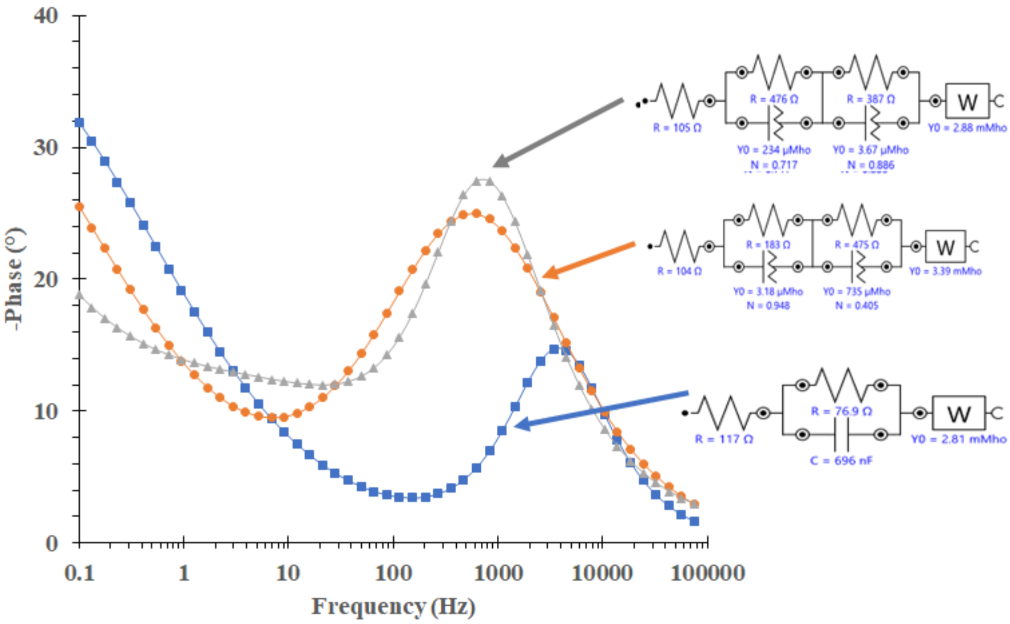

Figure S2. Bode phase plots and Randles' equivalent circuit at biosensor assembly. Electrochemical assays for the construction of the biosensor by gold (Au) surface modification, in 5.0 × 10^{-3} M [Fe (CN)6 $]^{3-}$ and 5.0 × 10^{-3} M [Fe (CN)6$]^{4-}$ solution, prepared in phosphate buffer, pH 7.4. Bode phase plot and the Randles' equivalent circuit were fitted to the EIS data of the Nyquist plot and are shown in Figure 5b.
